# Photobiomodulation With 976 nm Diode Laser Enhances Osteoblastic Differentiation of Saos‐2 Cells Cultured on Collagen Membranes

**DOI:** 10.1002/cre2.70333

**Published:** 2026-04-28

**Authors:** Escobar Lina M., Bendahan Zita, Pinzón Paula, Grajales Marggie, Baldion Paula

**Affiliations:** ^1^ Grupo de Investigaciones Básicas y Aplicadas en Odontología, IBAPO Facultad de Odontología Universidad Nacional de Colombia Bogotá Colombia; ^2^ Unidad de Manejo Integral de Malformaciones Craneofaciales UMIMC, Facultad de Odontología Universidad El Bosque Bogotá Colombia; ^3^ Laser Dentistry Master Program, European Program, EMDOLA University of Barcelona Barcelona Spain; ^4^ Departamento de Salud Oral, Facultad de Odontología Universidad Nacional de Colombia Bogotá Colombia

**Keywords:** collagen membranes, low‐level laser, osteoblastic differentiation, osteoblasts, photobiomodulation

## Abstract

**Objective:**

Laser photobiomodulation (PBM) influences cellular proliferation, morphology, and differentiation. Collagen membranes provide a bioactive scaffold that supports osteoblastic behavior; however, the effects of PBM on preosteoblastic Saos‐2 cells cultured on these membranes remain unclear. This study aimed to evaluate the impact of PBM on the proliferation, morphology, and differentiation of Saos‐2 cells grown on collagen membranes.

**Material and Methods:**

Saos‐2 cells were cultured either on collagen membranes (Jason, Straumann) or directly on plates. Experimental groups included: Control (Saos‐2), Saos‐2+ Jason, Saos‐2+Jason+Laser (976 nm diode), Saos‐2+Laser, and Saos‐2+Jason+ODM (osteogenic differentiation medium). Cell proliferation was assessed with the resazurin assay on days 2, 5, and 7. Morphology was analyzed by scanning electron microscopy (SEM). Reactive oxygen species (ROS) were quantified using DCFH‐DA, ATP production by luminescence assay, and osteogenic markers (RUNX2 and BMP2) by RT‐qPCR.

**Results:**

PBM reduced proliferation in Saos‐2+Jason+Laser cells (49.5% on day 2) without affecting viability, suggesting differentiation rather than cytotoxicity. SEM revealed increased cell size and membrane blebbing in irradiated membrane cultures, whereas non‐irradiated cells showed fewer extensions. PBM significantly upregulated RUNX2 and BMP2 expression on day 7, accompanied by elevated ROS and ATP levels, indicating enhanced metabolic activity and osteogenic induction in the Saos‐2+Jason+Laser group.

**Conclusions:**

PBM with a 976 nm diode laser induces significant cellular responses in Saos‐2 cells, characterized by reduced proliferation and enhanced osteoblastic differentiation, particularly when cultured on collagen membranes. These effects appear to be mediated by elevated ROS and ATP levels and by morphological characteristics consistent with differentiation. The findings highlight the potential of PBM as an adjunctive strategy for bone tissue regeneration and its promising application in regenerative dentistry, especially in combination with biomaterials such as collagen membranes.

## 1 Introduction

Tissue engineering aims to understand the structure and function of both normal and pathological tissues, as well as to develop biological substitutes capable of restoring, maintaining, or improving functionality (O'Brien 2011). Within its interdisciplinary framework, one of its fundamental bases is the use of porous three‐dimensional scaffolds, which function as temporary platforms to facilitate tissue regeneration. These scaffolds can be seeded with cells and enriched with growth factors or subjected to both biophysical and photochemical stimuli such as low‐level laser therapy (LLLT), which has emerged as a promising tool to modulate cellular processes and promote photobiomodulation (PBM) and tissue regeneration without generating significant thermal effects (Zhao et al. 2016; Zhu et al. 2017).

Laser PBM has demonstrated beneficial effects in promoting bone regeneration and modulating cellular activity. Previous studies have shown that laser treatment can stimulate both proliferation and differentiation of various cell types, as well as promote the expression of growth factors involved in tissue repair and regeneration (Pires Oliveira et al. 2008). At the cellular level, it induces an increase in mitochondrial size (Silveira et al. 2009), mitochondrial number, and membrane potential, leading to alterations in the ADP:ATP ratio, nucleotide activation, chromatin rearrangement, and increased mitochondrial protein synthesis (de Freitas and Hamblin 2016; Zheng and Yang 2021; Pchelin et al. 2023). Among the diverse applications of PBM, its effect on preosteoblastic cells cultured on collagen membranes has been of particular interest in in vitro studies (Renno et al. 2010). Collagen membranes provide three‐dimensional support that mimics the natural extracellular matrix, offering a favorable environment for cell adhesion, proliferation, and differentiation. Hence, they have emerged as one of the primary supporting biomaterials utilized in tissue engineering for reconstructive therapies because of its exceptional biocompatibility and biodegradability (Dos Santos et al. 2020). Combining laser stimulation with such biomaterials allows for the evaluation of synergistic effects that could optimize bone regeneration for clinical applications (Renno et al. 2010; Zhao et al. 2016; Zhu et al. 2017).

Research on the effects of PBM on preosteoblastic cell proliferation and differentiation has yielded promising outcomes. Stimulation with a He‐Ne laser (632 nm) significantly increases alkaline phosphatase activity and enhances the expression of osteopontin and bone sialoprotein in a human osteoblast cell line, suggesting stimulation of osteogenic differentiation (Stein et al. 2005).

Moreover, PBM has been reported to induce the formation of reactive oxygen species (ROS), which act as second messengers in multiple cellular signaling pathways, including activation of transcription factors such as HIF‐1α and upregulation of growth factors such as Vascular Endothelial Growth Factor (VEGF) and Transforming Growth Factor Beta (TGF‐β) (Amaroli et al. 2021; Bai et al. 2021). These mechanisms are fundamental to osteogenesis and angiogenesis—two interrelated processes essential for bone regeneration.

The combination of LLLT with collagen membranes therefore represents an attractive experimental approach for exploring how PBM can influence extracellular matrix organization and mineralization, both of which are critical for the formation of functional bone tissue. The interaction between the biophysical stimuli provided by the laser and the biochemical properties of collagen membranes may enhance therapeutic efficacy, opening new perspectives for their application in bone tissue engineering. However, despite the progress in this field, little is known about the specific effect of LLLT on Saos‐2 cells, particularly when cultured on collagen membranes.

Through an in vitro experimental approach, this study aims to provide scientific evidence supporting the use of this therapeutic combination in bone regeneration. Therefore, the objective of this work was to determine the effects of laser PBM on the proliferation, morphology, and differentiation of Saos‐2 preosteoblastic cells cultured on collagen membranes.

## 2 Materials and Methods

### 2.1 Treatment of Saos‐2 Preosteoblastic Cells With Low‐Power Laser

This quantitative in vitro experimental study analyzed preosteoblastic Saos‐2 cells (ATCC, HTB‐85) after approval by the institutional ethics committee (B CIEFO.OOS‐A‐2025). Laser PBM was applied to Saos‐2 cell cultures using a Woodpecker diode laser (Guilin Woodpecker Medical Instrument Co., Guangxi, China) operating at a wavelength of 976 nm. Prior to laser stimulation, the device was calibrated with an OPHIR NOVA II optical power meter (P/N 7Z01550; Ophir Optronics, Jerusalem, Israel). The laser characteristics and exposure parameters are summarized in Tables 1 and 2, and are defined following the standardized reporting protocol for laser parameters proposed by Jenkins and Carroll (2011).

**Table 1:** Technical specifications of the laser device.

Characteristic	Description
Wavelength (λ)	976 ± 20 nm^a^
Manufacturer	Woodpecker DBA
Model	LX 16 Plus
Place of manufacture	Guangxi, China
Active medium	GaAlAs semiconductor diode
Light beam delivery	Optical fiber (BT‐8 tip)
Operating mode	Continuous wave (CW), L1 mode
Beam profile	Gaussian
Maximum output power	976 nm: 7.0 W

^a^ Aiming beam 650 ± 20 nm, power 5 mW (diode laser).

**Table 2:** Laser application parameters.

Parameter (unit)	976 nm setting
Light beam output area (cm^2^)	0.38
Application surface (cm^2^)	1.00
Surface area per well^a^ (cm^2^)	0.32
Power density (W/cm^2^)	0.53
Output power (W)	0.20
Exposure time (s)	50
Energy density (J/cm^2^)	26.31
Energy per point (J)	10
Total energy per well^a^ (J)	10
Application points per well^a^	1
Application technique	Fixed‐point, manual support
Application distance^a^ (cm)	1.1
Number of applications	Single dose

^a^ Calculations performed for 96‐well cell culture plates.

**Figure 1:**
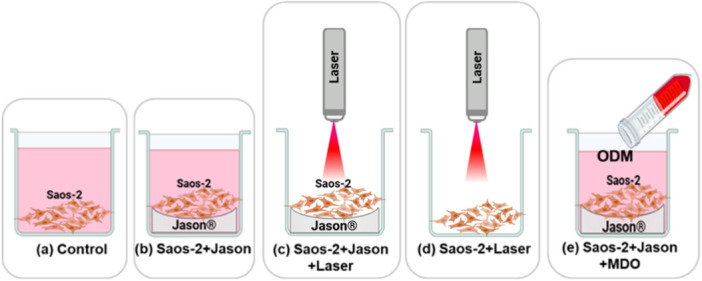
Experimental groups used in the study. (a) Control: Saos‐2 cells seeded without collagen membrane (Jason, Straumann, Basel, Switzerland) and without laser treatment or osteogenic differentiation medium (ODM); (b) Saos‐2+Jason: cells seeded on Jason collagen membranes; (c) Saos‐2+Jason+Laser: Saos‐2 cells seeded on Jason membranes and irradiated with a 976 nm laser; (d) Saos‐2+Laser: Saos‐2 cells treated with a 976 nm laser without seeding on membrane; and (e) Saos‐2+Jason+ODM: Saos‐2 cells seeded on Jason membranes and treated with ODM.

The applied laser power was 0.2 W, and the delivered energy dose was calculated per square centimeter (cm^2^). Parameter selection was based on previous studies by our research group (Garzón et al. 2022; Escobar et al. 2024, 2025). Each experimental condition received a single laser application. The irradiation distance was measured from the laser beam exit aperture to the bottom of the culture well, ensuring that the laser tip did not contact the wells surface to avoid contamination (Tables 1 and 2). To evaluate the effects of PBM, cells were seeded in multiwell plates and allocated into experimental groups shown in Figure 1. In certain groups, cells were cultured on commercial Jason collagen membranes (Straumann, Basel, Switzerland), composed of native porcine pericardium collagen. Additionally, an osteogenic differentiation medium (ODM) was used as a positive control. The ODM consisted of Dulbecco's Modified Eagle Medium (DMEM) supplemented with 10% fetal bovine serum (FBS), 100 U/mL penicillin, 100 µg/mL streptomycin, 0.1 µM dexamethasone (Sigma‐Aldrich, St. Louis, MO, USA), 5 mM β‐glycerophosphate (Santa Cruz, CA, USA), and 50 µg/mL ascorbic acid (Sigma‐Aldrich).

### 2.2 Determination of Cell Viability and Proliferation

Cell viability was evaluated under different experimental conditions (Figure 1) to analyze the cellular response to collagen membranes and their interaction with LLLT using a 976 nm diode laser. The assessment was performed through the resazurin reduction assay, which quantifies metabolic activity as an indicator of viable and metabolically active cells. For this purpose, Saos‐2 cells were seeded onto Jason collagen membranes (Straumann) placed in 96‐well plates at a density of 2 × 10^3^ cells/well. After an initial attachment period, the corresponding samples were subjected to laser irradiation with a 976 nm diode laser at a power output of 0.2 W for 50 s, except for the negative control group, which consisted of cells not exposed to the PBM protocol.

Prior to irradiation, both the plate lids and culture medium were removed aiming to prevent interferences with light transmission and avoid undesirable scattering, ensuring a more accurate laser–cell interaction. The culture medium was removed only during the 50‐s laser irradiation period, after which fresh medium was immediately added to prevent cell dehydration and death.

**Figure 2:**
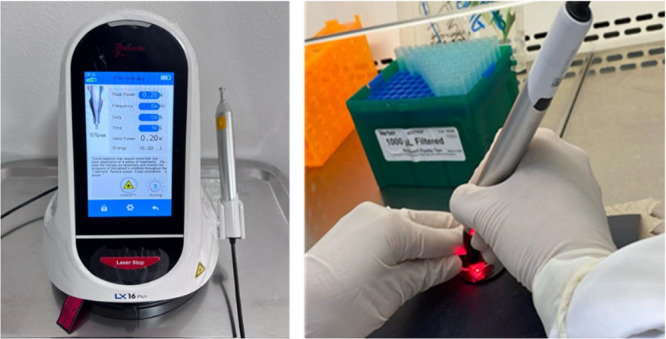
Application of laser PBM to Saos‐2 preosteoblastic cells. Saos‐2 cells were seeded in multiwell plates and treated according to the established experimental conditions. Prior to laser exposure, the culture medium was removed to ensure unobstructed light transmission. A single laser application was performed while the plate was covered with a black opaque sheet to prevent scattering of the emitted laser light into adjacent wells. Laser irradiation was delivered at a wavelength of 976 nm using an LX 16 Woodpecker diode laser device (Guilin Woodpecker Medical Instrument CO).

To guarantee precise laser targeting and avoid beam scattering into adjacent wells, empty wells were situated between those carrying cells, and each plate was covered with a black opaque sheet featuring a circular aperture that allowed light to reach only the designated well (Figure 2).

After incubation for the designated time points (2, 5, and 7 days), the culture medium was removed and a 10% v/v resazurin solution (7‐Hydroxy‐3H‐phenoxazin‐3‐one 10‐oxide), prepared from a 44 μM stock, was added to each well. The plates were then incubated for 3 h in a humidified atmosphere containing 5% CO_2_ at 37°C. Following incubation, fluorescence intensity was measured at an excitation wavelength of 535 nm and emission at 595 nm, using a microplate reader (Infinite M200, Tecan; Männedorf, Switzerland) controlled by Tecan i‐control software (Tecan Group Ltd., Männedorf, Switzerland). The percentage reduction of resazurin to resorufin was determined by comparing each experimental group with the untreated control, which was considered as 100% cell viability at each evaluation time point.

### 2.3 Evaluation of Morphological Changes in Saos‐2 Cells Cultured on Collagen Membranes and Treated With Laser

Preosteoblastic Saos‐2 cells were seeded onto Jason collagen membranes (Straumann) placed in 96 well plates at a density of 2 × 10^3^ cells/well. After cell attachment, cultures were subjected to PBM with a 976 nm diode laser at a power output of 0.2 W for 50 s. The negative control group consisted of cells not exposed to PBM. After 7 days of culture, cells were fixed with 4% paraformaldehyde and 2.5% glutaraldehyde for 1 h at room temperature, followed by three rinses with 1× phosphate‐buffered saline (PBS). A secondary fixation was performed with 1% osmium tetroxide (OsO_4_) for 1 h to preserve cellular ultrastructure. Samples were then dehydrated through a graded ethanol series (50%, 70%, 90%, and 100%) for 20 min at each concentration, followed by complete drying in a desiccator. Subsequently, specimens were sputter‐coated with gold/palladium alloy and examined for morphological characteristics using scanning electron microscopy (SEM) on a Vega‐TEScan microscope (Tescan USA Inc., Warrendale, PA, USA) operated at 20 kV under high‐vacuum mode. Images were acquired at different magnifications (×500, ×2000, and ×10,000) to evaluate changes in cell morphology, surface topography, and adhesion patterns on collagen membranes.

### 2.4 Assay for Detection of Reactive Oxygen Species

The generation of ROS in preosteoblastic Saos‐2 cells, cultured with or without Jason collagen membranes and subjected to PBM with a 976 nm diode laser at 0.2 W for 50 s, was evaluated using the 2′,7′‐dichlorofluorescein diacetate (DCF‐DA) assay (ab113851, Abcam, Cambridge, UK), following the protocol described by Wang and Joseph (1999). ROS detection was based on the probe 2′,7′‐dichlorodihydrofluorescein diacetate (H_2_DCF‐DA), which is deacetylated by intracellular esterases and subsequently oxidized by ROS to 2′,7′‐dichlorofluorescein (DCF), a fluorescent compound whose emission intensity can be quantified and is proportional to intracellular ROS levels.

For the experiment, 5 × 10^3^ cells per well were seeded in 96‐well plates and incubated with 25 μM H_2_DCF‐DA for 45 min at 37°C in the dark. After incubation, excess probe was removed by washing with PBS, and cells were immediately subjected to PBM under the specified parameters. To ensure accurate laser targeting and prevent light scattering into adjacent wells, the plate lid was removed, and the plate was covered with a black opaque sheet containing a circular aperture, allowing laser exposure exclusively within the region of interest.

Fluorescence was measured using a Tecan Infinite 200 Pro microplate reader equipped with Tecan i‐control software (Tecan Group Ltd.) at 485 nm excitation and 535 nm emission wavelengths. The fluorescence intensity, expressed in relative fluorescence units, was quantified and compared among the following groups: control (no laser exposure), laser‐treated cells, and cells cultured on collagen membranes with or without laser treatment. All analyses were performed in three independent experiments (*n* = 3), and results were normalized to the control group to determine relative changes in ROS production.

### 2.5 Detection of Cellular ATP Levels

Intracellular adenosine triphosphate (ATP) levels in preosteoblastic Saos‐2 cells cultured with or without Jason collagen membranes (Straumann) and subjected to PBM with a 976 nm diode laser at 0.2 W for 50 s, were determined using a luminescent ATP detection assay kit (ab113849, Abcam). This assay is based on the luciferase–luciferin bioluminescent reaction, in which light emission is directly proportional to the amount of ATP present in the sample. For the assay, 5 × 10^3^ cells per well were seeded in 96‐well microplates. After cell attachment, the designated groups were subjected to PBM. Before laser exposure, culture medium and plate lids were removed to avoid interferences with light transmission. During PBM, the plates were covered with a black opaque sheet containing a circular aperture to restrict the laser beam exclusively to the target area. As a positive control, an ATP standard curve was prepared by adding 90 μL of cell culture medium and 10 μL of ATP standard solution per well (final volume: 100 μL). For the measurement of intracellular ATP, 50 μL of detergent solution was added to each test well to induce cell lysis. Plates were sealed and shaken for 5 min at 700 rpm, followed by the addition of 50 μL of substrate solution (luciferase and luciferin). After gentle mixing, the plates were incubated in the dark for 10 min to stabilize the luminescent signal.

Luminescence was then measured using a Tecan Infinite 200 Pro microplate reader equipped with Tecan i‐control software (Tecan Group Ltd., Männedorf, Switzerland). Results are expressed as mean ± standard deviation (SD) of three independent experiments (*n* = 3) and reported as relative ATP levels in relative light units normalized to the non‐irradiated control group.

### 2.6 Evaluation of Osteoblastic Differentiation Gene Expression in Saos‐2 Cells Stimulated With PBM

Preosteoblastic Saos‐2 cells were seeded at a density of 2 × 10^3^ cells per well in 96‐well plates and allowed to adhere for 24 h under standard culture conditions. Subsequently, cells were treated with a 976 nm diode laser at 0.2 W for 50 s. Following laser treatment, total RNA was extracted using the Quick‑RNA MicroPrep Kit (Ref. #R1051, Zymo Research, Irvine, CA, USA) according to the manufacturer's instructions. RNA yield and purity were determined spectrophotometrically (A₂₆₀/A₂₈₀ ratio). Complementary DNA (cDNA) was synthesized by reverse transcription using the ProtoScript II First Strand cDNA Synthesis Kit (Ref. #E6560L, New England BioLabs, Ipswich, MA, USA).

Briefly, RNA sample (final concentration 200 μg/μL) were denatured with a random hexamer primers mixture (60 μM) for 5 min at 65°C in a BioRad T100 Thermal Cycler (Bio‑Rad Laboratories, Hercules, CA, USA). The reaction mixture contained the ProtoScript II (2×) mix with deoxynucleotide triphosphates (dNTPs), an optimized reaction buffer, ProtoScript II Reverse Transcriptase and a murine‑derived RNase inhibitor. Reverse transcription was performed at 25°C for 5 min, followed by 42°C for 60 min, as recommended by the manufacturer. A negative control was included by replacing the RNA template with nuclease‐free water, while maintaining all other reaction components and conditions identical to those of the experimental samples. The expression of Runt‐related transcription factor 2 (RUNX2) and bone morphogenetic protein 2 (BMP2) genes was analyzed to assess osteoblastic differentiation. Quantitative real‐time PCR (RT‐qPCR) was performed using the Luna Universal qPCR Master Mix (New England BioLabs), which employs the SYBR Green I fluorescent dye, and quantified on a CFX96 Real‐Time PCR System (Bio‐Rad Laboratories). Glyceraldehyde‐3‐phosphate dehydrogenase (GAPDH) served as the reference (housekeeping) gene for normalization.

Amplification was carried out under the following cycling conditions: initial denaturation at 95°C for 3 min, followed by 50 cycles of 95°C for 10 s, 60°C for 30 s, and 72°C for 20 s, and a final melting curve analysis consisting of 65°C for 5 s and 95°C for 5 s to confirm amplicon specificity. Primer sequences and their characteristics are summarized in Table 3. PCR amplification efficiencies were calculated using LinRegPCR software (Academic Medical Center, AMC, Amsterdam, Netherlands), and relative gene expression levels were determined according to the method described by Schefe et al. (2006), using GAPDH as the internal reference gene.

**Table 3:** Primer characteristics used in this study.

Gene	Forward primer (5′→3′)	Reverse primer (3′→5′)	Amplicon size (bp)
RUNX2	CATCTAATGACACCACCAGGC	GCCTACAAAGGTGGGTTTGA	168
BMP2	CGAAACACAAACAGCGGAAAC	GCCACATCCAGTCGTTCCA	97
GAPDH	GAAGGTGAAGGTCGGAGTC	GAAGATGGTGATGGATTTC	226

Abbreviations: BMP2: bone morphogenetic protein 2; GAPDH: glyceraldehyde‐3‐phosphate dehydrogenase; RUNX2: runt‐related transcription factor 2.

### 2.7 Data Analysis

All statistical analyses were performed using IBM SPSS Statistics version 27 (IBM SPSS, Chicago, IL, USA). Descriptive statistics, including measures of central tendency (mean) and dispersion (SD), were calculated for all response variables across experimental groups. The normality of data distribution was assessed using the Shapiro–Wilk test, and homogeneity of variances was verified with Levene's test. For data meeting parametric assumptions, comparisons among groups were conducted using one‐way analysis of variance followed by Tukey's honestly significant difference post hoc test. When normality or homoscedasticity assumptions were not satisfied, non‐parametric analyses were applied using the Kruskal–Wallis test followed by Dunn's multiple comparison procedure. All data are presented as mean ± SD. Data are expressed as mean ± SD, and differences were considered statistically significant at a *p*‐value ≤ 0.05.

## 3 Results

### 3.1 Effect of PBM on the Proliferation of Pre‐Osteoblastic Cells Seeded With and Without Collagen Membrane

**Figure 3:**
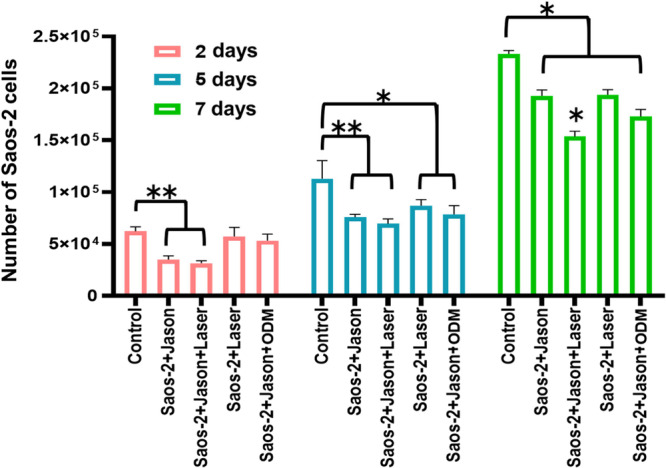
Changes in Saos‑2 cell number induced by PBM treatment. A significant reduction in Saos‑2 cell number was observed in cultures seeded on Jason (Straumann) collagen membranes, both treated and untreated with a 976 nm diode laser at 0.2 W for 50 s, beginning at day 2. At days 5 and 7, all experimental groups exhibited a significant decrease in cell number compared with the control group (cells not subjected to the PBM protocol) within the same incubation period. Statistical analysis was performed among the five experimental groups for each time point, considering *p* ≤ 0.05 (*) and *p* ≤ 0.01 (**) as statistically significant. Data are presented as mean ± SD. Collagen membrane (Jason), osteogenic differentiation medium (ODM).

Pre‐osteoblastic Saos‐2 cells cultured on Jason collagen membranes (Straumann), with or without PBM using a 976 nm diode laser, exhibited a significant reduction in cell number as early as 2 days post‐treatment, compared to the negative control. This decrease in cell number persisted throughout the 7‐day assessment period. In contrast, cells cultured without membranes but exposed to laser stimulation (Saos‐2+Laser), and those seeded on collagen membranes and treated with ODM (Saos‐2+Jason+ODM), showed a significant reduction in cell number beginning on day 5 following PBM protocol. The most pronounced reduction in cell proliferation was observed in the Saos‐2+Jason+Laser group, with decreases of 49.5% on 2 days, 38.4% on 5 days, and 34% on 7 days post‐laser exposure, compared to the control (Figure 3). Importantly, the reduction in cell number was attributed to decreased proliferative activity, as no significant increase in cell death was detected by trypan blue exclusion assay (data not shown). These findings suggest that PBM with a 976 nm diode laser may modulate the proliferative behavior of pre‐osteoblastic cells, particularly when cultured on collagen membranes, potentially promoting early differentiation rather than cytotoxic effects.

### 3.2 Morphological Changes in Saos‐2 Cells Cultured on Collagen Membranes and Treated With PBM

**Figure 4:**
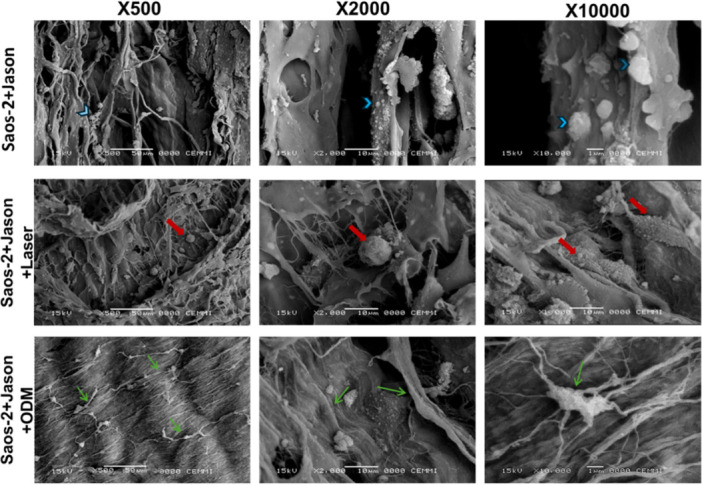
Morphological changes in Saos‐2 pre‐osteoblastic cells cultured on collagen membranes under different treatments. Representative scanning electron microscopy (SEM) micrographs (×500, ×2000, ×10,000) showing morphological variations in Saos‐2 cells after 7 days of culture on Jason collagen membranes (Straumann). Cells cultured without PBM (Saos‐2 + Jason) exhibited a flattened morphology with few cytoplasmic extensions (blue arrowheads). Cells treated with PBM using a 976 nm diode laser at 0.2 W for 50 s (Saos‐2 + Jason + Laser) appeared enlarged and showed membrane blebbing and irregular surface contours (red arrows). Cells grown on membranes in osteogenic differentiation medium (ODM) (Saos‐2 + Jason + ODM) displayed elongated cytoplasmic projections and a well‐organized filamentous network across collagen fibers (green arrows). PBM: photobiomodulation; ODM: osteogenic differentiation medium.

Morphological alterations in Saos‐2 pre‐osteoblastic cells cultured on Jason collagen membranes (Straumann) and treated under different experimental conditions were analyzed by SEM at magnifications of ×500, ×2000, and ×10,000 (Figure 4).

Cells cultured on collagen membranes without laser exposure (Saos‐2 + Jason) exhibited a flattened morphology with limited cytoplasmic extensions and poor spreading over the membrane surface. The collagen fibers remained clearly visible, and cells appeared sparsely distributed, indicating minimal interaction with the substrate. In contrast, cells subjected to PBM with a 976 nm diode laser (Saos‐2 + Jason + Laser) showed notable morphological modifications. These included an increase in cell size, membrane blebbing, irregular surface contours, characteristics of cytoskeletal remodeling associated with cell activation or early differentiation. The cells appeared more integrated into the collagen matrix, suggesting enhanced adhesion and metabolic activity. Meanwhile, cells cultured on collagen membranes in the presence of ODM (Saos‐2 + Jason + ODM) displayed numerous elongated cytoplasmic projections and dense filamentous networks extending across the collagen fibers. These structures are indicative of active matrix interaction and osteogenic differentiation, resembling the morphology of mature osteoblasts.

Collectively, SEM observations revealed a progressive morphological transition from poorly spread, flattened cells (non‐irradiated control) to enlarged and metabolically active cells with membrane protrusions (laser‐treated) and finally to elongated, highly interconnected cells (ODM group). These findings support the hypothesis that PBM at 976 nm promotes cytoskeletal reorganization and differentiation rather than proliferation or cytotoxic effects in Saos‐2 cells cultured on collagen membranes.

### 3.3 Changes in the Expression of Osteoblastic Differentiation Genes in Saos‑2 Cells Stimulated With Collagen Membrane and PBM

**Figure 5:**
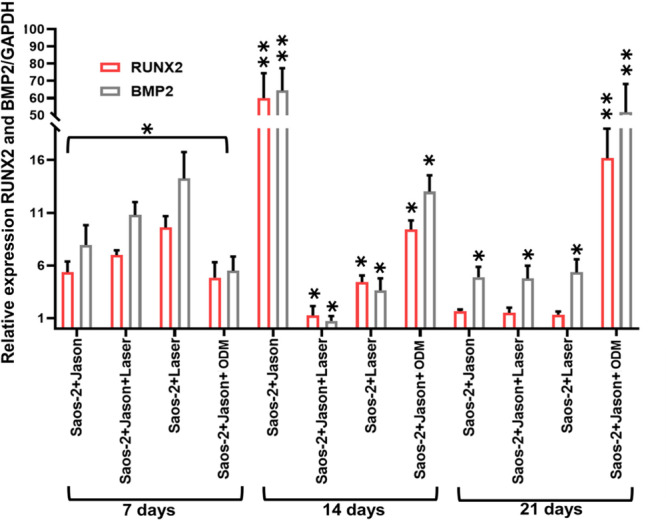
Quantification of RUNX2 and BMP2 gene expression. Quantification of RUNX2 and BMP2 gene expression was performed in Saos‑2 cells treated with a 976 nm laser for 7, 14, and 21 days. Data are presented relative to GAPDH gene expression and to the control group, which consisted of Saos‑2 cells cultured without Jason membrane, laser irradiation, or osteogenic differentiation medium (ODM). Statistical significance was set at *p* ≤ 0.05 (*) and *p* ≤ 0.01 (**). Values are expressed as mean ± SD. Collagen membrane: Jason (Straumann).

To evaluate the potential of PBM to induce osteoblastic differentiation, the expression levels of key osteogenic markers—RUNX2 and BMP2—were quantified by RT‑qPCR technique (Figure 5).

Exposure of Saos‑2 cells to the 976 nm diode laser for 50 s resulted in a marked and statistically significant upregulation of both RUNX2 and BMP2 expression as early as 7 days post‑treatment, with the most pronounced increase observed in the Saos‐2+Jason+Laser group. At 14 days, gene expression remained elevated in cells cultured on Jason collagen membranes (Saos‑2+Jason) and those treated with ODM (Saos‐2+Jason+ODM). Laser‐treated groups (Saos‐2+Laser and Saos‐2+Jason+Laser) also exhibited enhanced expression at this time point, although to a lesser extent than at day 7.

By 21 days, a significant and sustained upregulation of RUNX2 and BMP2 persisted only in the Saos‑2+Jason+ODM group, suggesting active osteogenic differentiation. In contrast, the remaining groups displayed a decrease in RUNX2 expression over time, whereas BMP2 expression remained moderately elevated, indicating the maintenance of osteogenic signaling rather than continued transcriptional activation of RUNX2.

### 3.4 Production of Reactive Oxygen Species (ROS) and Adenosine Triphosphate (ATP) in Saos‐2 Cells Stimulated With PBM and Cultured on Collagen Membranes

**Figure 6:**
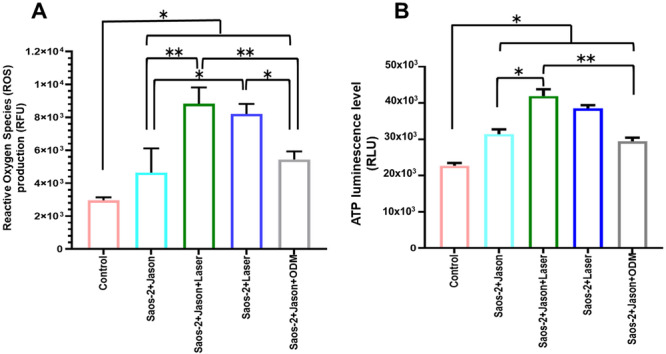
Detection of ROS and ATP levels in Saos‐2 cells subjected to PBM and cultured on collagen membranes. (A) ROS production was quantified using the 2′,7′‐dichlorofluorescein diacetate (DCF‐DA) assay in cells irradiated with a 976 nm diode laser at 0.2 W for 50 s and expressed in relative fluorescence units. (B) ATP levels were determined by luminescent assay, reported as relative light units. Collagen membrane: Jason (Straumann); ODM: osteogenic differentiation medium; Control: Saos‐2 cells cultured without membrane and without laser stimulation. Values represent the mean ± SD from three independent experiments (*n* = 3). Statistical significance: *p* ≤ 0.05 (*) and *p* ≤ 0.01 (**).

A significant increase in ROS production was observed in Saos‐2 cells cultured on Jason collagen membranes (Saos‐2+Jason), in cells exposed to PBM while seeded on collagen membranes (Saos‐2+Jason+Laser), in those irradiated without membranes (Saos‐2+Laser), and in cells cultured on membranes with ODM (Saos‐2+Jason+ODM). The most pronounced elevation in ROS levels occurred in the Saos‐2 +Jason+Laser and Saos‐2+Laser groups compared with the control group (Saos‐2 cells cultured without membrane or laser exposure) (Figure 6A). As shown in Figure 6B, ATP levels also increased significantly, particularly in cells treated with PBM—both with and without collagen membranes (Saos‐2+Jason+Laser and Saos‐2+Laser)—relative to the control and to non‐irradiated groups (Saos‐2+Jason and Saos‐2+Jason+ODM). The parallel increase in ROS and ATP levels suggests that PBM at 976 nm for 50 s enhances mitochondrial metabolic activity and cellular energy production, consistent with early differentiation processes in pre‐osteoblastic cells.

## 4 Discussion

This study evaluated the effects of PBM using a 976 nm diode laser on Saos‐2 preosteoblastic cells cultured on collagen membranes. A significant decrease in cell proliferation was observed in Saos‐2 cells cultured on Jason collagen membranes and exposed to a 50‐s PBM protocol, without evidence of cytotoxicity, suggesting that the inhibitory effect is related to cellular differentiation processes, rather than cell death. PBM + Jason membranes induced morphological changes consistent with differentiation, increased ROS and ATP production, and an early upregulation of the osteogenic genes RUNX2 and BMP2. Together, these findings support the potential of combining laser therapy and collagen membranes as a strategy to promote osteoblastic differentiation. Although several studies have reported that PBM can stimulate osteoblastic cell proliferation (Peplow et al. 2010; Oliveira et al. 2016; Laffitte et al. 2024), even in the presence of osteogenic factors (Bloise et al. 2013), the present work demonstrated a significant reduction in cell number, beginning at 2 days post‐treatment and persisting through day 7. This decrease was most evident in the Saos‐2+Jason+Laser group, with reductions of 49.5% (day 2), 38.4% (day 5), and 34% (day 7). Differences in cell types, laser parameters, and the interaction between PBM and the collagen matrix could account for this discrepancy. The three‐dimensional architecture of the membrane may modulate cellular behavior, favoring differentiation over proliferation. Moreover, previous research has shown that specific levels of ROS generation can transiently arrest the cell cycle, promoting a shift toward differentiation (Valko et al. 2007).

Furthermore, morphological changes observed in PBM‐treated cells, included cytoskeletal reorganization and the formation of membrane blebs, structures that facilitate directed migration of mesenchymal cells to bone‐forming regions. This migratory capacity is essential during intramembranous ossification, where progenitor cells must proliferate and differentiate into osteoblasts (Dalby et al. 2007). Cell migration is typically enhanced during the early stages of bone formation and declines as cells become more adherent (Sliogeryte et al. 2014). In contrast, cells cultured with ODM displayed longer cytoplasmic projections, suggesting stronger adhesion and cytoskeletal maturation associated with the differentiation process. Unfortunately, a comparison with prior research discussing these structural changes in Saos2 cells induced by PBM under similar application parameters to those of this current study is unfeasible, as such features have not been deemed relevant.

Collagen membranes, such as Jason (Straumann), provide bioactive microenvironments that promote cell adhesion and osteoblastic differentiation (Dos Santos et al. 2020; Varela et al. 2023). Previous studies have shown that similar membranes stimulate the expression of RUNX2, ALP, and BMP2 in human osteoblast cultures (Li et al. 2025). Their ability to retain growth factors and provide a three‐dimensional structure resembling native bone matrix enhances their osteoinductive potential (Jung et al. 2011; Fujioka‐Kobayashi et al. 2017). Conversely, Varela et al. (2023) demonstrated in their rat study that the application of collagen membranes led to a 78% decrease in the transmission of 808 nm laser across surgical defects, thereby attenuating the PBM effect on bone regeneration compared to the irradiated group lacking a membrane. To avoid this reported interference, the present study positioned the collagen membrane at the bottom of each well containing Saos‐2 cells. Nevertheless, since a potential interaction between the membrane and the laser could not be entirely excluded, the output power was slightly increased by 0.2 W to compensate for this possible effect.

In parallel, although a sham‐irradiated control device was not incorporated into the experimental design, several methodological precautions were implemented to minimize thermal and handling‐related artifacts during laser application. The PBM protocol employed a 976 nm diode laser using carefully selected parameters—0.20 W output power, 50 s exposure time, and an energy density of 26.31 J/cm^2^ at a power density of 0.53 W/cm^2^—applied at a fixed point with a manual holder and a standardized application distance of 1.1 cm. These irradiation conditions were deliberately maintained well below the thermal risk thresholds described by Cronshaw et al. (2023), who observed significant temperature elevations (> 3°C) only at energy densities exceeding 100 J/cm^2^, particularly under higher power outputs (> 1 W) and prolonged exposure durations (> 120 s). Moreover, as highlighted by Zein et al. (2018), maintaining irradiance levels below 0.75 W/cm^2^ is recommended to prevent unwanted photothermal effects while preserving PBM efficacy; notably, the parameters used in the present study conform to this guideline. All experimental groups underwent identical handling procedures, including membrane placement and laser positioning (with the laser switched off in non‐irradiated controls), thereby minimizing potential mechanical confounders. Collectively, the use of low irradiance, short exposure times, and tightly controlled experimental conditions strongly suggests that the biological effects observed are attributable to PBM rather than nonspecific photothermal responses.

Growing evidence supports the use of laser PBM to activate signaling pathways involved in osteogenesis. PBM has been reported to upregulate key osteogenic markers, including RUNX2, BMP2, and COL1A1, and to enhance mineralization in osteoprogenitor cell models (Patrocínio‐Silva et al. 2014). Nevertheless, the biological response to PBM appears to be dependent on both the irradiation parameters and the differentiation stage of the cells, as the effects of 660 nm stimulation on osteoblastic cells have been shown to vary accordingly (de Araújo et al. 2024). While the majority of PBM studies have traditionally focused on wavelengths ranging from 650 to 810 nm, more recent investigations have explored longer wavelengths within the near‐infrared spectrum, including 976 nm, reporting promising osteogenic outcomes (Gutiérrez et al. 2021). In this context, Hanna et al. (2019) demonstrated that 980 nm laser irradiation (0.9 W/cm^2^, ~55 J/cm^2^) significantly increased RUNX2 expression and alkaline phosphatase (ALP) activity in MC3T3‐E1 preosteoblastic cells, and similar osteogenic responses have since been described in human cell models exposed to 980 nm lasers (Etemadi et al. 2024; Ma et al. 2025), in agreement with the present findings. Importantly, wavelength‐specific biological effects are closely linked to the spectral properties and absorption coefficients of intracellular chromophores. The binding affinity of cytochrome‐c oxidase (COX) has been shown to progressively decrease from 450 nm to wavelengths above 900 nm, whereas absorption by water and lipids increases with longer wavelengths. In addition, wavelengths in the range of 905–908 nm have been reported to modulate cellular responses in a dose‐ and condition‐dependent manner (Sommer 2019; Agas et al. 2021).

Within this framework, one limitation of the present study is the use of Saos‐2 osteosarcoma‐derived cells, which differ genetically and phenotypically from primary human osteoblasts or mesenchymal stem cells (MSCs). However, Saos‐2 cells are widely recognized as a valid in vitro model for early‐stage investigations of osteoblastic differentiation and biomaterial–cell interactions due to their stable phenotype and consistent expression of key osteogenic markers such as ALP, type I collagen, and osteocalcin (Rodan et al. 1987; Murray et al. 1987; Pautke et al. 2004). Their high reproducibility and reduced donor variability make them particularly suitable for controlled assessments of PBM parameters and enhance the comparability of results across laboratories (Wilkesmann et al. 2020). Previous studies have demonstrated that PBM using various laser wavelengths can effectively modulate osteogenic responses in Saos‐2 cells, including increased expression of ALP and RUNX2, especially during the early phases of differentiation (Chellini et al. 2010; Ghidini et al. 2021). While studies employing primary osteoblasts or MSCs are essential to increase translational relevance, complementary evidence from our previous work using human dental pulp stem cells (hDPSCs) showed that laser PBM promoted cell proliferation, redox balance, and osteogenic commitment (Escobar et al. 2024). Together, these complementary cellular models support the validity of the current findings and underscore the need for future studies incorporating multiple human‐derived cell systems to further expand the clinical significance of PBM in bone regeneration strategies.

In the present study, 976 nm laser irradiation of Saos‐2 preosteoblastic cells seeded on collagen membranes produced a significant increase in osteogenic gene expression and mitochondrial activity, supporting the hypothesis that this wavelength can induce cellular changes favorable for bone regeneration. These findings agree with those reported by Agas et al. (2021), who showed that 980 nm laser exposure modulates preosteoblastic metabolism via activation of the PI3K/Akt/Bcl‐2 pathway, depending on dose. However, under their experimental conditions, no direct effects on differentiation markers were observed, underscoring the importance of energy density and irradiation parameters in PBM efficacy.

In the context of osteogenesis, PBM‐induced mitochondrial activation precedes the expression of key osteogenic markers such as RUNX2, ALP, and BMP2 (Li et al. 2017; Smith and Eliseev 2021), indicating that mitochondrial metabolism not only supports cell survival but also drives phenotypic commitment to the osteoblastic lineage.

Overall, our results confirm that PBM with 976 nm laser, particularly when combined with collagen membranes, promotes early osteoblastic differentiation by enhancing RUNX2 and BMP2 expression, ROS generation, and ATP synthesis. This suggests activation of mitochondrial metabolism and related cell signaling pathways, consistent with previously proposed PBM mechanisms (de Freitas and Hamblin 2016). The increases in ROS and ATP observed align with the mitochondrial model of PBM action, in which light absorption by COX (complex IV of the mitochondrial respiratory chain) firstly stimulates oxidative phosphorylation (Leyane et al. 2021). As a result, ATP production increases, improving cellular metabolism, while controlled ROS levels function as second messengers for intracellular signaling (Leyane et al. 2021). COX, along with porphyrins and heme proteins, acts as a primary photoacceptor, initiating a cascade of photochemical and photophysical reactions (Avci et al. 2013). Although Karu (2008) reported that COX exhibits maximal activity within the 580–700 nm wavelength range, biological effects have also been detected at longer wavelengths, such as the 976 nm used in this study. These responses may be linked to elevated intracellular Ca^++^ and ATP levels resulting from the modulation of voltage‐sensitive calcium channels, which promote the release of nitric oxide. Acting as a secondary messenger, nitric oxide (NO) subsequently activates signaling pathways including MAP‐kinase, ERK1/2, β‐catenin, and Wnt, as well as bone matrix–related genes, thereby enhancing cellular proliferation, viability, and differentiation (Amaroli et al. 2016; Karu et al. 2005; Karu 2008).

Energy absorption by COX stimulates oxidative phosphorylation, which increases ATP synthesis and promotes moderate ROS generation and cyclic AMP accumulation, both of which regulate intracellular signaling (Wu et al. 2014; Mosca et al. 2019). Moreover, PBM can promote NO dissociation from COX, reversing its inhibitory effect and modulating oxidative stress at the mitochondrial membrane (Karu et al. 2005).

These mechanisms collectively activate signaling cascades, gene transcription, and protein synthesis associated with cell proliferation, migration, and differentiation. The resulting increase in ATP availability enhances ion transport (Ca²⁺, K⁺, Na⁺) and activates transcription factors such as NF‐κB and MAPK, key regulators of regenerative processes (Leyane et al. 2021). These mechanisms are consistent with our findings, in which PBM with 976 nm laser treatment enhanced ATP production and osteogenic differentiation in Saos‐2 cells.

Collectively, these results reinforce the role of PBM as a key modulator of mitochondrial metabolism, with downstream effects on the activation of osteogenic signaling pathways, in line with previous evidence showing that PBM stimulates mitochondrial activity, increases ATP synthesis, and promotes the release of growth factors (Leyane et al. 2021). These growth factors, in turn, activate membrane receptor–mediated signaling pathways that upregulate genes involved in cell viability, migration, and differentiation—processes that are essential for tissue repair and bone regeneration. Although late‐stage functional endpoints of osteogenesis, such as ALP activity, collagen deposition, or mineralized nodule formation, were not evaluated in the present study, the experimental approach was deliberately focused on early‐stage molecular and mechanistic events. The upregulation of key osteogenic transcription factors, particularly RUNX2 and BMP2, represents a reliable early indicator of osteoblastic commitment, as these markers precede extracellular matrix maturation and mineralization. Moreover, the primary objective of this work was to investigate the combined effects of collagen membranes and 976 nm PBM on early cellular responses, with particular emphasis on mitochondrial activation, ATP production, and controlled ROS modulation, which constitute critical upstream events that prepare cells for subsequent matrix production and mineralization.

Taken together, our findings suggest a synergistic interaction between the collagen membrane and 976 nm PBM, generating a microenvironment that supports cell adhesion, mitochondrial activation, and osteoblastic differentiation. In vivo, the combination of PBM with autologous grafts and membranes has been shown to enhance tissue regeneration by stabilizing graft material and accelerating bone formation (Dos Santos et al. 2020; Freitas et al. 2018). Freitas et al. reported that intraoperative PBM using an 808 nm GaAlAs laser significantly accelerated bone repair in critical defects, with the greatest bone regeneration observed when combined with a collagen membrane. The authors attributed this synergy to PBM‐induced ATP production, osteoblastic activation, collagen fiber organization, angiogenesis stimulation, and anti‐inflammatory gene regulation. Collagen membranes, in turn, provide a biocompatible scaffold that maintains space for bone regeneration, minimizes graft resorption, and enhances graft stability.

This integrated approach demonstrates both biological efficacy and clinical potential, offering practical advantages such as fewer treatment sessions, shorter healing times, and improved accessibility compared with protocols requiring multiple postoperative PBM applications. While extended culture periods and functional mineralization assays are warranted to confirm mature osteogenesis, the present findings provide a strong mechanistic and translational foundation supporting the use of PBM combined with collagen membranes in bone regeneration strategies.

Overall, the results of this study support the clinical potential of PBM with a 976 nm diode laser as an adjuvant for guided bone regeneration, particularly when combined with collagen membranes. This combination may enhance the predictability of implant, periodontal, and reconstructive procedures by promoting cell maturation and tissue integration. Nevertheless, controlled clinical trials are warranted to validate its long‐term efficacy and safety.

## 5 Conclusions

PBM with 976 nm diode laser applied to Saos‐2 preosteoblastic cells cultured on collagen membranes induced a decrease in cell proliferation without cytotoxic effects and promoted significant activation of osteoblastic differentiation. This response was evidenced by the upregulation of RUNX2 and BMP2 gene expression, morphological changes, and increased intracellular ROS and ATP levels. Together, these findings suggest that PBM enhances mitochondrial and transcriptional activity associated with osteogenic commitment. The synergistic interaction between the 976 nm laser and the collagen membrane suggests that this combined approach may serve as an effective strategy to stimulate early osteoblastic differentiation and bone tissue regeneration, with potential translational applications in dental and orthopedic regenerative therapies. However, caution should be exercised when extending these findings to clinical settings, where the complex interactions among diverse cell types and extracellular matrix components differ substantially from the controlled conditions of in vitro experiments.

## Author Contributions

**Escobar Lina M.:** conceptualization, funds acquisition, study design, experimentation, data analysis and interpretation, manuscript drafting, final approval of the manuscript for submission. **Bendahan Zita:** experimentation, data analysis and interpretation, manuscript drafting, final approval of the manuscript for submission. **Pinzón Paula:** conceptualization, data curation, methodology, supervision, writing – review. **Grajales Marggie:** study design, data analysis and interpretation, manuscript drafting, final approval of the manuscript for submission. **Baldion Paula:** conceptualization, data curation, formal analysis, investigation, methodology, supervision, validation, visualization, roles/writing – original draft, writing – review and editing.

## Ethics Statement

The study protocol was approved by the Institutional Ethics Committee (Act B.CIEFO‐005‐A‐2024).

## Conflicts of Interest

The authors declare no conflicts of interest.

## Data Availability

Data supporting this study are available from the corresponding author upon request.
